# The Soil in Tuberculosis

**Published:** 1914-12

**Authors:** 


### The Soil in Tuberculosis.

It is becoming every day more and more apparent to thinking physicians that a more serious study of the soil in tuberculosis is necessary. For over thirty years sanitarians have chiefly directed their efforts for the prevention of tuberculosis to the dèstruction of the tubercle bacillus. Less attention has been given to the scientific treatment of the actively tuberculous. Practically no study has been given the morbid soil so ripe for invasion.

With the one thought in view, preventative medicine has largely satisfied itself with the study of the biology of the bacillus. In the laboratory we have determined the chemical structure of the organism and the elements that make it more tenacious of life than many other bacteria. We have even determined the length of time it may remain virulent under various external conditions. We have likewise determined the value of the various physical and chemical agents used to destroy the organism. We are fully informed as to the various channels of infection. We understand more or less clearly the mechanism of infection and how the normal body defends itself against invasion.

But all the information has not enabled us to kill one iota of the myriads of bacilli expelled by the hundreds of thousands of active victims of the white plague. Can we even say that tuberculosis is any less prevalent now than it was twenty years ago? It seems that, after three decades of bacilli hunting, we ought to direct our efforts to eradicate the disease along other lines. This does not mean that we should abate our efforts to destroy the bacillus. Great good has undoubtedly been accomplished, and to this we must hold fast.

The thousands upon thousands of children of tuberculous parents, the overworked and ill nourished children of the poor, and the victims of pernicious habits and other chronic diseases all furnish a fertile soil in which the tubercle bacillus may thrive. This soil has never received the study or treatment that it merits. Every child whose stock of vitality is lowered by parental disease is predisposed to infections of many kinds, and probably over 50 per cent contract tuberculosis in time, if other diseases do not terminate their existence too soon. Every ill nourished and uncared-for child is susceptible to the same infection. Probably 30 per cent or more of all children under fourteen are infected and only await the proper moment for active tuberculosis to develop. We probably underestimate both the extent and significance of latent tuberculosis. We use the term predisposition as if it were a definite morbid process, and yet we do not know why the predisposed are less resistant than normal children. We have not even outlined a rational treatment for such, and can not until we understand the process better. We know in part only what takes place in early infection.

The time has come for a broader work in tuberculosis, and every agency should be employed to combat this universal scourge. We should seek to increase the natural resistance, to prevent early infection, to understand the processes of predisposition, latent infection and active infection, and to develop a rational treatment.

To increase the natural powers of resistance, the marriage of the actively tuberculous should be discouraged. This should be done by statutory enactment requiring a medical certificate of health before issuing a license to marry. Few will deny the duty of the State, or question her right. It should be equally apparent that for the actively tuberculous to bring children into existence is to increase the burden of the State.

To prevent early infection, we should separate the child from the actively tuberculous parent at the earliest possible moment. This may seem cruel, but it is not nearly so cruel as it is to allow the child to become infected and in turn infect others, thus keeping up a continuous line of disease. Humanity demands a wholesome environment for the young.

To understand the processes of predisposition, latent infection and active infection, a laboratory for the study of tuberculosis is necessary. The one million infected school children will furnish an inexhaustible supply of material. There is certainly some factor present or absent in the predisposed that modern science can determine, rendering such individuals susceptible to infection. In the case of infection, we should determine what takes place in the infected organism. If metabolism is increased and demineralization occurs regularly, it is important that the fact should be established.

To establish a rational treatment is the aim of all investigation. Such a treatment must be based on known processes and conditions. At the present moment our treatment of tuberculosis is neither satisfactory nor complete. We simply know in part, and we treat in part.

THEO. Y. HULL, M. D.

---

Below will be found an appeal for funds for the afflicted physicians in Belgium.

Dr. H. Edwin Lewis, of *American Medicine*, is to be commended for having undertaken this great humanitarian movement. Let us respond today to this appeal. Times may be hard, but you can spare something. Let us make a good showing in this as in all things that we undertake. Every State in the Union will be invited to contribute. Will our Texas physicians do their share? I answer yes, for I know no State in the Union has a set of physicians who are broader, more generous or liberal than those of dear old Texas.

FOR THE AFFLICTED PHYSICIANS OF BELGIUM.

The physicians of Belgium are in direst need. Starvation and cold will soon cause the most terrible suffering unless steps are at once taken to save them and their dependents.

The great urgency of the situation has made it necessary to hasten our efforts and a committee organized under the auspices of *American Medicine* has undertaken the collection of a fund for Belgian physicians.

Every medical man, every medical journal, every kindhearted person in America is urged to contribute—if only to the amount of 25 cents. Every penny will help.

Contributions may be sent directly to the fund for Belgian physicians, care of *American Medicine,* 18 East Forty-first Street, New York City, or if preferred to TEXAS MEDICAL JOURNAL.

The all important thing is to send your contribution, however humble it may be, at the earliest possible moment. Winter is close at hand, and the suffering our sorely afflicted brethren are sure to undergo will be appalling unless we who are placed in happier circumstances do something *now—right away—today* to lighten their burden of sorrow and distress.

Only a small contribution is solicited from each individual, but if a goodly number will respond it is certain that a sum will be realized that will save our Belgian colleagues from the horrors of famine and the cold. Send in your contribution today.

Address, Fund for Belgian Physicians, care of *American Medicine,* 18 East Forty-first Street, New York City, or if you prefer the editor of this journal will receive your contribution and forward it to the committee in charge of this movement.

